# Simulation Study on the Application of the Generalized Entropy Concept in Artificial Neural Networks

**DOI:** 10.3390/e20040249

**Published:** 2018-04-03

**Authors:** Krzysztof Gajowniczek, Arkadiusz Orłowski, Tomasz Ząbkowski

**Affiliations:** Department of Informatics, Faculty of Applied Informatics and Mathematics, Warsaw University of Life Sciences-SGGW, Nowoursynowska 159, 02-787 Warsaw, Poland

**Keywords:** artificial neural network, simulation study, generalized entropy

## Abstract

Artificial neural networks are currently one of the most commonly used classifiers and over the recent years they have been successfully used in many practical applications, including banking and finance, health and medicine, engineering and manufacturing. A large number of error functions have been proposed in the literature to achieve a better predictive power. However, only a few works employ Tsallis statistics, although the method itself has been successfully applied in other machine learning techniques. This paper undertakes the effort to examine the q-generalized function based on Tsallis statistics as an alternative error measure in neural networks. In order to validate different performance aspects of the proposed function and to enable identification of its strengths and weaknesses the extensive simulation was prepared based on the artificial benchmarking dataset. The results indicate that Tsallis entropy error function can be successfully introduced in the neural networks yielding satisfactory results and handling with class imbalance, noise in data or use of non-informative predictors.

## 1. Introduction

Artificial neural networks (ANN) are flexible and powerful statistical learning models used in many applications. They have been extensively and successfully applied in areas such as signal processing, pattern recognition, machine learning, and system control in a number of real-world problems including business, medicine or engineering [1,2,3,4,5]. Several features of artificial neural networks make them very popular and attractive for practical applications. First, they possess an ability to generalize with high tolerance to incomplete or noisy data; Second, neural networks are non-parametric thus they do not require *a priori* assumptions about the data distribution; Third, they are good approximators, able to model continuous function to a desired accuracy, assuming no limitations for hidden neurons or layers.

From a pattern recognition perspective, the neural network needs to determine the required mapping from input to output variables in order to solve the classification or the regression problem. The main question in neural networks training is to find the correct values for the weights between the input and output layer using a supervised learning paradigm. During the training process the difference between the prediction made by the network and the correct value for the output is calculated, and the weights are updated accordingly to minimize the error. The form of the error function is one of the factors in the weight update process. For the successful application it is important to train the network with an error function that reflects the objective of the problem. The mean square error (MSE) is the most commonly used function, although it has been suggested in the literature that it is not necessarily the best function to be applied, especially in classification problems [6,7,8]. Therefore, a number of alternative error functions have been proposed and the maximum likelihood (cross entropy) function was particularly reported as a more appropriate function for classification problems [7,8].

In this paper we undertake the effort to examine an alternative error function which is the q-generalized function based on Tsallis statistics. In particular, the properties of the function and its impact on the neural network classification accuracy are analyzed.

To the best of our knowledge the proposed error function was not examined before in the context of the neural network learning and taking into account different aspects of underlying data specifics. For this reason we have simulated a benchmarking dataset to facilitate identification of the strengths and weaknesses of the proposed error function applied to neural network training. Simulated benchmarking is used to confirm that a new method successfully runs as expected and to prove its reliability under different data conditions [9]. Testing new approach can involve a wide range of evaluation metrics, including prediction accuracy, computational and model complexity, which are then verified under different dataset assumptions. To meet the requirement of the throughout diagnosis of the proposed error function applied to neural network we have tested its performance assuming that classification results are tested against the presence of: (1) linear and nonlinear signals; (2) non-informative predictors; (3) correlation among the predictors; (4) class imbalances.

The rest of this paper is organized as follows: the Section 2 consists of an overview of the similar research problems. In the Section 3 the theoretical framework of the generalized entropy is presented. The Section 4 describes theoretical aspects of the artificial neural networks and the details of q-generalized error function applied. Next, in the Section 5, the simulation details for the benchmarking dataset are brought. The Section 6 deals with the experiments and presents the discussion of the results. The paper ends with concluding remarks in the Section 7.

## 2. Literature Review on Similar Problems

The research stream on neural networks is still impressively growing and the literature around this field is covering various aspects of network configurations. While the method itself becomes a more and more substantial part of the state-of-the-art automatic pattern recognition systems applicable in a variety of fields, different research questions arise regarding such elements as the network architecture or the fundamentals of the training process. Usually, the works include modifications and improvements of the neural network structure, weights initialization [10], weights updating procedure [11], error functions [12,13] and activation functions [14,15]. The training process of artificial neural networks usually requires that users specify an error function to adapt the network parameters (weights) to meet certain model performance criteria. The error measure is very important and in certain circumstances it is essential for achieving satisfactory results. To train feedforward neural networks different error measures have been applied including the mean-square error measure (with modifications) and cross-entropy, as the most popular ones [8]. It can be depicted that the true posterior probability is reaching a global minimum for both the cross-entropy and squared error criteria, as summarized in [8,16]. Therefore, in the theory, ANN can be well trained by minimizing both of the functions, as long as it is able to approximate the true posterior distribution arbitrarily close. When it comes to the modelling a continuous distribution, squared error is bounded and therefore, the optimization is more robust to outliers than minimization of cross-entropy. However, in practice, cross-entropy converge quickly resulting better quality in terms of classification accuracy. For this reason, squared error has lost on popularity over the last years [8,17].

When it comes to applications under the nonextensive statistics with Tsallis distributions, called q-distributions, formed by maximizing Tsallis entropy with certain constraints—such distributions have applications in physics, astronomy, geology, chemistry and finance [18]. However, these q-distributions remain largely unnoticed by the computational science, with only a few works applying them to ANNs, not necessary as the error functions [19]. For instance, [19] introduces q-generalized RNNs (random neural network) for classification where parametrized q-Gaussian distributions are used as activation functions. These distributions arise from maximizing Tsallis entropy and have a continuous real parameter q—the entropic index—which represents the degree of nonextensivity.

The other application by Fernández-Navarro et al. [20] proposes a radial basis function neural network, called the q-Gaussian RBFNN, that reproduces different radial basis functions by means of a real parameter q. The results show that the q-Gaussian model can be considered competitive in comparison to other classification methods.

Similarly, Tinos et al. [21] propose q-Gaussian function as a radial basis function in radial basis function network for pattern recognition problems with positive outcome. The use of q-Gaussian RBFs allows to modify the shape of the RBF by changing the real parameter q, and to employ radial units with different RBF shapes in a same network structure.

In order to address the identified literature gap, in this paper, we present an investigation on the properties of the q-entropic error criteria for training of ANNs. The theoretical analysis of the error bounds was supported by experimental evaluation of the networks taking into account their classification accuracy based on the simulated benchmarking dataset.

Benchmarking datasets typically can be either real-world data or artificially generated with known, underlying patterns. While the real-world benchmarks can describe many different domains, from a scientific perspective, many of the benchmarks freely available in the repositories can have very similar meta-features like the number of observations, number of variables, number of classes, presence of missing data, and similar signal to noise ratios as reported in [22]. Although they are representative of different real-world phenomena, they may not represent a diverse structure of data science problems. For this reason, designing a specific dataset remains a valid point if the goal is to conduct a comprehensive analysis of the proposed approach being able to identify both strengths and shortcomings. We believe the simulated dataset proposed in this work enables us to draw meaningful conclusions.

## 3. Theoretical Framework of the Generalized Entropy

Entropy is a measure of unpredictability of the state, or equivalently, of its average information content. The concept of information entropy was introduced by Claude Shannon [23] which is defined as:

(1) $$H_{S}=-\overset{n}{\underset{i=1}{\sum }}t_{i}logt_{i},$$

where $t_{i}$ is the probability of occurrence of an event $x_{i}$ being an element of a random variable X that can take values $x_{i},\ldots ,x_{n}$. The value of the entropy depends on two parameters: (1) disorder (uncertainty) and it is maximum when the probability $t_{i}$ for every $x_{i}$ is equal; (2) the value of n. Shannon entropy assumes a tradeoff between contributions from the main mass of the distribution and the tail. To control both parameters a generalization was proposed by Tsallis with the goal to provide a foundation for nonextensive statistical mechanics [24]:

(2) $$H_{T_{q}}=\frac{1}{q-1}\left(1-\overset{n}{\underset{i=1}{\sum }}t_{i}^{q}\right).$$

With Shannon entropy, events with high or low probability have equal weights in the entropy computation. However, using Tsallis entropy, for q>1, events with high probability contribute more than low probabilities for the entropy value. Therefore, the higher is the value of q, the higher is the contribution of high probability events in the final result.

Since artificial neural network requires error function that accepts target variable and predicted value, to measure the distance between two distributions the Kullback-Leibler divergence can be introduced. Generally K-L divergence measures the inefficiency of assuming that the distribution is $\left\{y_{i}\right\}$, when the true distribution is $\left\{t_{i}\right\}$:

(3) $$D_{KL}(t_{i}||y_{i})=\overset{n}{\underset{i=1}{\sum }}t_{i}log(t_{i}/y_{i}).$$

It should be noted that it is not a true “distance” measure between the two distributions since it is not symmetric, i.e., it does not satisfy the triangle inequality.

By the same token the mutual Tsallis entropy is a generalization of the K-L entropy, the latter being just a limiting case of the former for q→1. Mutual Tsallis entropy refers to two probability distributions $\left\{t_{i}\right\}$ and $\left\{y_{i}\right\}$, i=1 to n, over the same alphabet, and is defined as (see, e.g., [25]):

(4) $$H_{T_{q}}(t_{i}||y_{i})=\frac{1}{1-q}\left(1-\overset{n}{\underset{i=1}{\sum }}t_{i}^{q}y_{i}^{1-q}\right).$$

In the limit q=1, one has $H_{T_{1}}(t_{i}||y_{i})=\sum_{i=1}^{n}t_{i}log(t_{i}/y_{i})$, i.e., the Kullback-Leibler relative entropy. For any value of q≠0, the Tsallis relative entropy $H_{T_{q}}(t_{i}||y_{i})$ given by the above equation vanishes if and only if $t_{i}=y_{i}\;$ for all i=1 to n. For any q>0, the Tsallis relative entropy $H_{T_{q}}(t_{i}||y_{i})$ is always nonnegative. In this regime of q>0, the Tsallis relative entropy $H_{T_{q}}(t_{i}||y_{i})$ behaves much like the conventional Kullback-Leibler relative entropy, yet in a generalized form identified by the additional parameter q.

## 4. Theoretical Framework of the Artificial Neural Network

Artificial neural networks are computing systems inspired by the biological neural networks that constitute animal brains. Such systems learn tasks by considering examples, generally without task-specific programming. In common ANN implementations, the synapse signal is a real number, and the output of each neuron is calculated by a non-linear function of the sum of its inputs. Neurons and synapses typically have weights that adjust as learning proceeds. The weights increase or decrease the strength of the signal that it sends across the synapse. 

**Figure 1:**
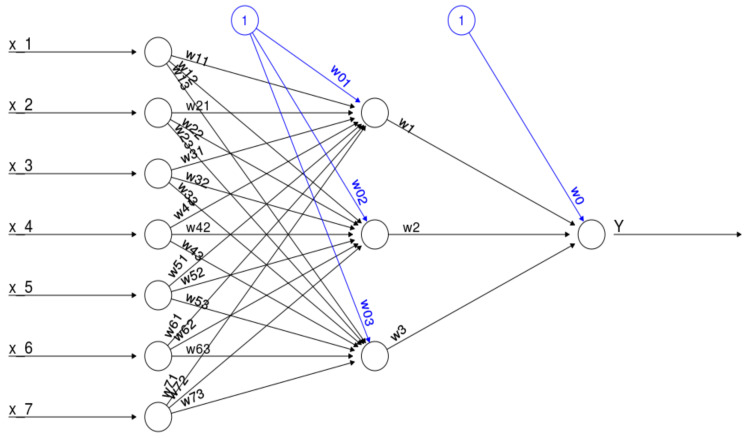
Example of an artificial neural network with seven input neurons (independent variables), one output neuron (target variable) and one hidden layer consisting of three hidden neurons.

Typically, neurons are organized in layers [26]. Different layers may perform different kinds of transformations on their inputs. Signals travel from the first (input), to the last (output) layer, possibly after traversing the layers multiple times. Figure 1 gives an example of a neural network (feedforward artificial neural network with initial random weights) which has seven input neurons (equal to the number of independent variables) and one output neuron (equal to the number of target variables) [27]. In general neural network with a one hidden layer consisting of J hidden neurons and p input neurons calculates the following function (please compare this equation with Figure 1):

(5) $$ANN=f\left(w_{0}+\overset{J}{\underset{j=1}{\sum }}w_{j}f\left(w_{0j}+\overset{p}{\underset{i=1}{\sum }}w_{ij}x_{i}\right)\right),$$

where $w_{0}$ denotes the synaptic weight of the intercept of the output neuron, $w_{0j}$ denotes the synaptic weight of the intercept of the j-th hidden neuron, $x_{i}$ denotes the i independent variable. Additionally, $w_{j}$ denotes the weight corresponding to the synapse starting at the j-th hidden neuron and leading to the output neuron and $w_{ij}$ denotes the weight of the i-th neuron from the input layer to the j neuron from the hidden layer [27]. Moreover, f denotes the activation function which is usually a bounded nondecreasing nonlinear and differentiable function such as the logistic function defined as:

(6) $$f\left(x\right)=\;\frac{1}{1+e^{-x}}.$$

Neural networks are fitted to the data by learning algorithms during a training process. These learning algorithms are characterized by the usage of a given output that is compared to the predicted output and by the adaptation of all parameters according to this comparison. The parameters of an artificial neural networks are their weights. The process stops if a pre-defined criterion is met (please refer to the Equation (19) in the Section 6.2). A commonly used learning algorithm is the resilient backpropagation algorithm which modifies the weights of a neural network in order to find a local minimum of the error function E [28]. Therefore, the gradient of the error function is calculated with respect to the following formula (all weights from neural network are stored in one vector containing (p+1)∗J+(J+1) weights):

(7) $$w_{k}^{\left(i+1\right)}=w_{k}^{\left(i\right)}-\eta_{k}^{\left(i\right)}\mathrm{sign}\left(\frac{\partial E^{\left(i\right)}}{\partial w^{\left(i\right)}}\right),$$

where k indexes the weights, i the iteration steps and $\eta_{k}$ learning rate which will be increased if the corresponding partial derivative keeps its sign. On the contrary, it will be decreased if the partial derivative of the error function changes its sign since a changing sign indicates that the minimum is missed due to a too large learning rate.

The error function being minimized by the optimization algorithm should accepts a vector of parameters, input data, output data, and should returns both, the current value of the error function and its gradients. Due to that the error function which implements the Tsallis entropy is defined as below [29]:

(8) $$E\left(t,y,q\right)=\frac{1}{1-q}\left(1-t^{q}y^{1-q}-{\left(1-t\right)}^{q}{\left(1-y\right)}^{1-q}\right),$$

where t and y stand for the true value and output of neural network respectively and q stands for the current value of the q-parameter. Moreover its gradient is defined as follows:

(9) $$\frac{\partial }{\partial y}E\left(t,y,q\right)={\left(-\left(y-1\right)y\right)}^{-q\;}\left(y^{q}{\left(1-t\right)}^{q}-{\left(1-y\right)}^{q}t^{q}\right).$$

**Figure 2:**
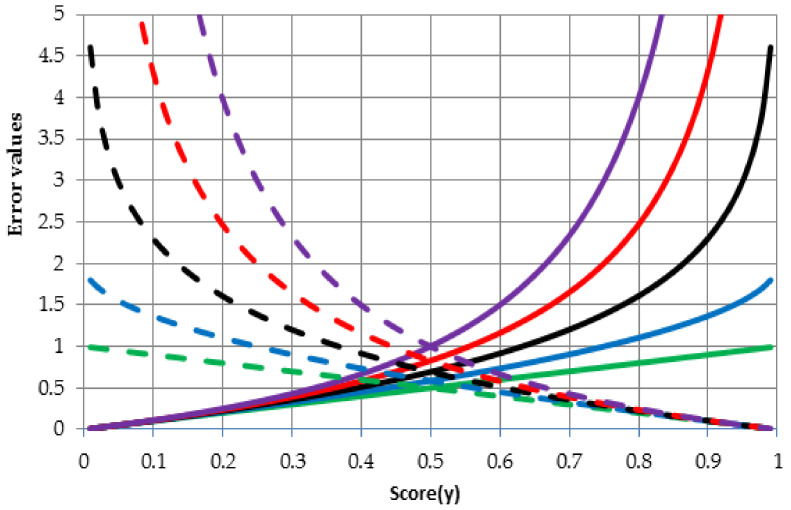
Error entropy values for different q values in terms of different score values.

Figure 2 shows the behavior of the error function for different values of parameter q and two possible values of target variable t i.e., 0 (solid lines) and 1 (dashed lines). Link between colors and values of the parameter q is as follows: 0—green, 0.5—blue, 1—black (in this case q is equal almost 1 because when q=1 formula (4) or (8) becomes the Kullback–Leibler relative entropy), 1.5—red, 2—purple. The solid lines show entropy values for the growing outcome (variable y from Equations (8) and (9)) of neural network in case if the true value is 0 (target variable t from Equations (8) and (9)). It can be noticed that if the outcome (score from neural network ranging between 0 and 1) tends to 1 then the error function increases, in other words this is undesirable situation for a given case. In contrast, the dashed lines show entropy values for the growing outcome in case if the true value is 1. Once again, entropy values increase when the neural network outcome is inconsistent with the true value. In general, behavior of the error function with q-parameter greater than 1 is more non-linear than behavior of error function based on the Shannon entropy (q≈1).

## 5. Simulation of the Artificial Dataset

In order to evaluate any sort of statistical method, it is recommended to perform a proper simulation study which can produce results where the true signal is known in advance. Good simulation system should test a few different aspects of classification tasks:

- linear and nonlinear independent variables;
- non-informative independent variables;
- correlation among the independent variables;
- class imbalances.

In this study simulation system models the log-odds of a binary event as a function of true signals using several additive sets of a few different types. To simulate the data, the *twoClassSim* function from *caret* package was used [30]. This function simulates dataset with two labeled classes, truly important independent variables and irrelevant independent variables.

The final dataset was simulated in several steps. In the first step, two multivariate standardized normal independent variables (denoted here as $N_{1}$ and $N_{2}$) were created with a Spearman correlation coefficient about 0.65. These variables change the log-odds using main effects and an interaction:

(10) $$intercept-4N_{1}+4N_{2}+2N_{1}N_{2}.$$

The intercept is a parameter that controls the amount of class imbalance. This is due to the fact that it has the biggest influence on probability defined in Equation (15) i.e., when it takes value close to 0 the overall probability is 0.5 and when decreasing to negative values the overall probability approaches to 1. Therefore, the probability of sampling positive class (denoted as 1) decreases from 0.5 to 0.05. In this simulation study different values of these parameters were used. This set of variables is highly required for the simulation purpose.

The second set consists of independent variables which are linear (denoted here as L) with coefficients that alternate signs and have values ranging from 2.5 to 0.025. Let us make an example, if there are four independent variables in this set, their contribution to the log-odds would be [30]:

(11) $$2.50L_{1}+1.75L_{2}+1.00L_{3}+0.25L_{4}.$$

Those variables are meaningful for our analysis and they will be used further.

In order to add some fluctuation to the log-odds, the third dataset containing nonlinear function of a single independent variable ranging between [0, 1] (called here NN) was created [30]:

(12) $$NN^{3}+2e^{-6{\left(NN-0.3\right)}^{2}}.$$

This single variable is meaningful for the simulation and will be used in the main analysis.

The fourth set of informative independent variables uses two more predictors (K and M) denoted as [30]:

(13) 2sin(KM).

Also, in this case those variables will be used in the main analysis.

All of these independent variables are added up to model the log-odds. This is used to derive the probability of a sample being in the first class and a random uniform number is used to actually make the assignment of the actual class. The target variable y is a Bernoulli random variable which takes the value 1 with probability p and the value 0 with probability q=1−p:

(14) Pr(y=1)=p=1−Pr(y=0)=1−q.

Altogether, for each data sample, the target variable is simulated from the Bernoulli distribution with the probability p derived from the inverse logit function:

(15) $$p=\frac{1}{1+e^{-\left(intercept-4N_{1}+4N_{2}+2N_{1}N_{2}+2.50L_{1}+1.75L_{2}+1.00L_{3}+0.25L_{4}+NN^{3}+2e^{-6{\left(NN-0.3\right)}^{2}}+2\sin\left(KL\right)\right)}}$$

For each simulation, the non-informative independent variables were added. These were random standard normal variables and were added to the data in two specified manners [30]:

- Fifth set with a specified number of uncorrelated independent variables (denoted as UC);
- Sixth set with a specified number of independent variables that follow a particular correlation structure (denoted as C).

**Figure 3:**
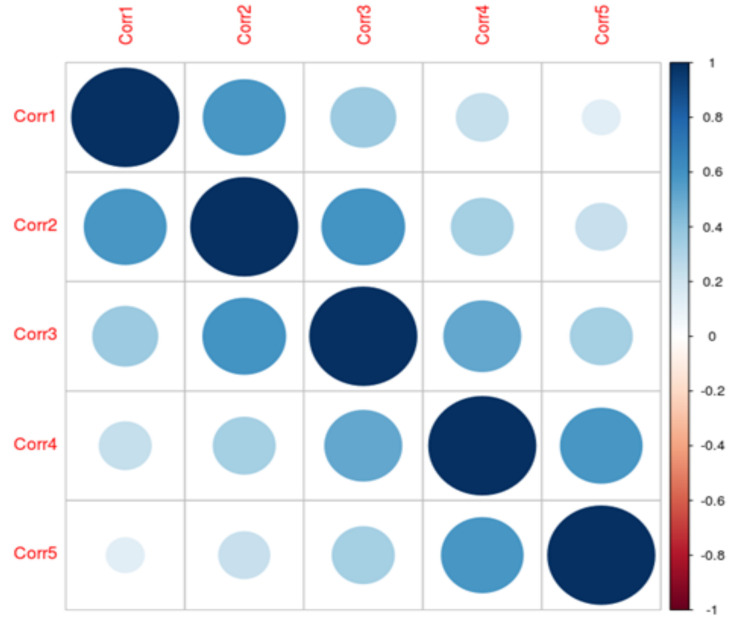
Correlation structure between independent variables from the sixth set.

In the second case the independent variables are auto-regressive 1 (AR(1)). While there is no time component to these data, this structure can be used to add independent variables of varying levels of correlation. For instance, if there are 5 independent variables and the correlation parameter is 0.6, then the correlation matrix would be as depicted in the Figure 3.

For AR(1), correlations decrease as the independent variables are further away from each other (in a proper order).

Altogether the whole settings of the simulation study can be summarized as follow:

- Separate training, validation and test sets were simulated, which contain approximately 1000 (50%), 600 (30%) and 400 (20%) observations respectively;
- The intercept parameter controls the overall level of class imbalance and was selected to yield a ratio of around:
  ⚬ 50% of the first class and 50% of the second class,
  ⚬ 40% of the first class and 60% of the second class,
  ⚬ 30% of the first class and 70% of the second class,
  ⚬ 20% of the first class and 80% of the second class,
  ⚬ 10% of the first class and 90% of the second class,
  ⚬ 5% of the first class and 95% of the second class,
- 2 meaningful independent variables were included ($N_{1}$ and $N_{2}$ variables from the first set);
- 3 meaningful independent variables were included (L variables from the second set);
- 1 meaningful independent variable was included (NN variable from the third set);
- 2 meaningful independent variables were included (K and M variables from the fourth set);
- 3 meaningless uncorrelated independent variables were created (UC variables from the second set);
- 3 meaningless correlated independent variables that follow AR(1) structure were simulated (C variables from the sixth set).

The whole simulation study is the combination of the aforementioned settings when the class imbalance ratio differs from 50–50% up to 5–95%. The choice of these ratios was dictated by the willingness to cover the whole spectrum of problems observed in practical applications. When the proportions of classes are quite similar the problem is relatively simple to model and most of the algorithms can be applied. However, if the class imbalance is observed (and this is very common situation) researchers have to perform additional actions such as over/under-sampling and application of the robust algorithms.

## 6. Numerical Experiment

### 6.1. Accuracy Measures

In order to evaluate the model performance, two measures were used: classification accuracy, sensitivity and area under the receiver operating characteristics curve (AUC). As far as binary classification is concerned, the models yield two classes, positive and negative, so there are possible four outcomes, as shown in Table 1 [31].

Based on the Table 1, the overall accuracy (AC) measure can be estimated, which is the proportion of the total number of predictions that were correct. It is estimating using the below equation:

(16) $$AC=\;\frac{TP+TN}{TP+FP+TN+FN}.$$

In order to construct the ROC curve and calculate the AUC value, two measures have to be determined i.e., false positive rate $Fpr=\frac{FP}{FP+TN}=1-Tnr$ and true positive rate $Tpr=\frac{TP}{TP+FN}$. These indicators can be estimated for different threshold values (in relation to the score from the neural network). An increase of the threshold from 0 to 1 will yield to a series of points (Fpr, Tpr) constructing the curve with Fpr and Tpr on the vertical and horizontal axes, respectively. Eventually the AUC measure is given by:

(17) $$AUC=\;\overset{1}{\underset{0}{\int }}ROC\left(u\right)du.$$

**Figure 4:**
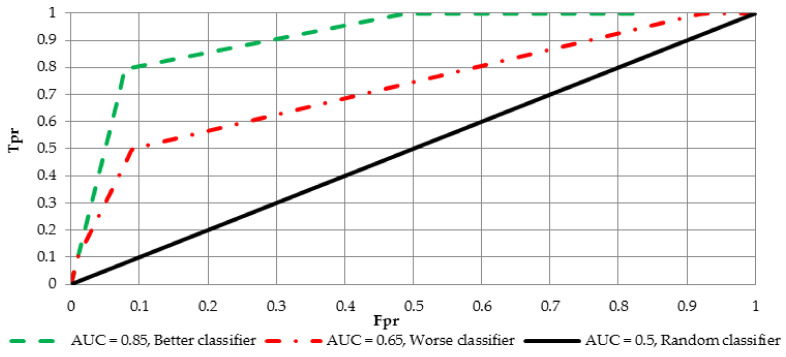
The ROC curve and its possible variants.

AUC measure has also another interpretation. Let $X_{n}$ and $X_{p}$ ale the labels for negative and positive cases, respectively. It could be observed that $AUC=P\left(X_{p}>X_{n}\right),$ which is the probability that in a randomly drawn pair of positive and negative cases the classifier probability is higher for the positive one. For the purpose of interpretation and comparison of multiple curves, two possible variants of the ROC curve are shown in Figure 4.

In order to benefit from the optimal score threshold with respect to the Equation (15) Youden’s J statistic [32] was employed. The optimal cut-off is the value that maximizes the distance to the diagonal line and is defined as:

(18) max(Tpr+(1−Fpr)).

### 6.2. Implementation

**Figure 5:**
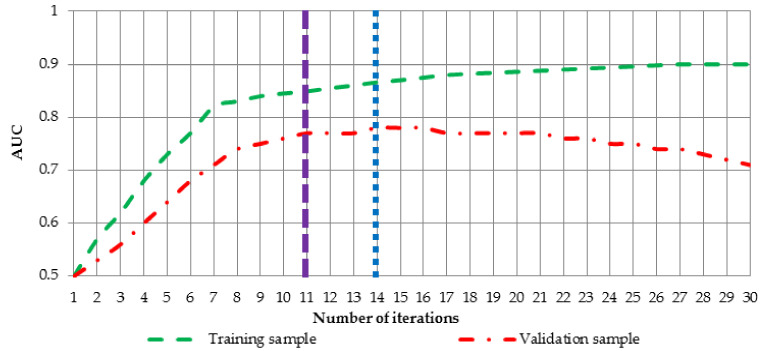
Example of AUC values for training and for validation sample with respect to the number of iteration.

Numerical experiment was conducted in *R* environment using computer equipped with Intel Core i5-2430M 2.4 GHz processor (2 CPU × 2 cores), 8 GB RAM and the Ubuntu 16.04 LTS operating system. In order to achieve neural networks that have good generalization abilities, special learning process incorporating AUC measure (this measure is commonly used to evaluate the quality of binary classification models) was performed. Therefore, to find the best parameters while training the neural networks, the following function was maximized:

(19) $$f\left(AUC_{T},AUC_{V}\right)=-w\left|AUC_{T}-AUC_{V}\right|+\left(1-w\right)AUC_{V}$$

where $AUC_{T}$ stands for the training error, $AUC_{V}$ for the validation error. Parameter w measures the weights of the first and the second term in the equation i.e., it controls what is more important during learning, stability of the neural network or small error on the unseen dataset. To give practical example of the behavior of the Equation (19), Figure 5 has been created.

Figure 5 shows AUC values for training (green dashed line) and validation (red dotted-dashed line) sample for ongoing learning process of the neural network. If for instance, weight parameter w takes value 0.2, learning process should be terminated after 14 iterations (blue dotted line) and if it takes value 0.7, optimal neural network is achieved after 11 iterations (dashed purple line).

In general, the whole learning process comprises of the following steps and parameters:

- Dataset was simulated in the way described in the Section 5, i.e., six different simulations were conducted, each for different class imbalance ratio;
- The resilient backpropagation algorithm with weight backtracking was used to calculate the neural network;
- Since the simulated dataset has 15 variables (14 independent variables and one target variable), the number of neurons in the hidden layer was iteratively increased from 1 to 15, in order to maintain triangle/rectangle like structure;
- Logistic function was used to activate all neurons in the neural network;
- The q-parameter was considered in the range between 0 and 2 by 0.1;
- The w parameter in the Equation (19), was sequentially increased from 0 to 1 by 0.1 (see below bullet);
- To avoid overfitting, after the completion of each learning iteration (with a maximum of 100 iterations), the neural network was checked for the error measure defined in Equation (19). At the end, the neural network characterized by the smallest error was chosen as the best model (proper end point of learning process);
- In order to achieve robust estimation of the neural networks error, sixteen different neural networks were learned with different initial weights vector (4 different random seeds) and for different datasets samples (4-times cross-validation). Final estimation of the error (for each q-value and for each number of hidden neurons) was computed as the average value over sixteen neural networks.

### 6.3. Results

In this section we describe several experiments aimed to test and to compare the performance of neural networks with regard to q-entropy cost function. To determine the relation between the ANN performance and the q-parameter values the following analysis were prepared:

- Training capability in terms of the number of iterations required and the number of hidden neurons;
- Model’s performance in terms of AUC values and classification accuracy for the test sample.

All aforementioned features were combined with q-values with respect of class imbalance ratio (six different cases) or w parameter (eleven different values) from Equation (19).

#### 6.3.1. Results for Different Class Imbalance Ratio

The first analysis was to examine the relation between q-parameter and ANN ability to reach convergence in terms of the training function maximization (19) while the w parameter was set at 0.5 (it is valid for cases in this subsection), what means that both components were treated equally.

**Figure 6:**
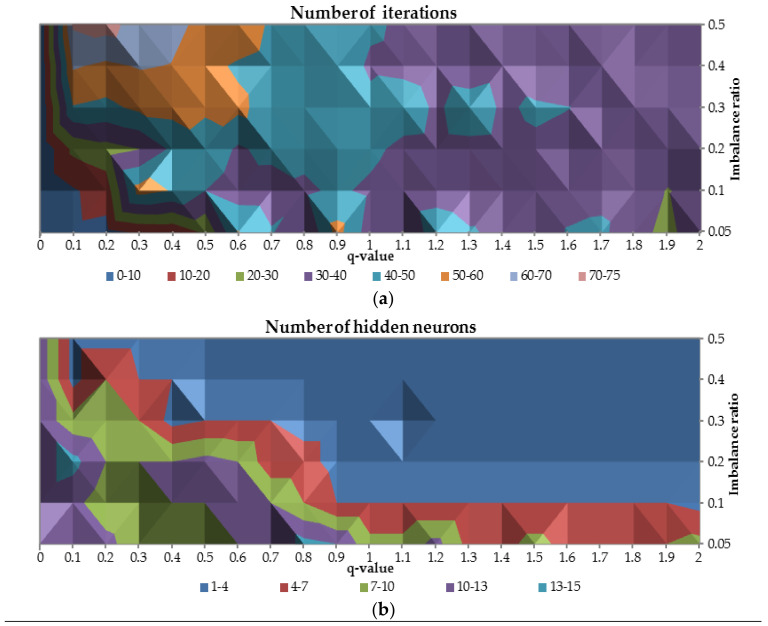
The relation between the number of iterations (**a**) and the number of hidden neurons (**b**) in terms of different q-values and class imbalance ratio.

Therefore, the number of iterations to gain the convergence and the number of hidden units were plotted against q-parameter and class imbalance ratio as presented in Figure 6. When the q is equal to 0 less than 10 iterations are needed to train the network for all cases with class imbalance ratio. For q-values between 0.1 and 0.7 and for class imbalance ratio less than 0.3, the number of iterations required is greater than 50. In the middle of the figure, for all the cases with class imbalance ratio, it occurred that number of iterations required is between 40 and 50. When the q-value increase the number of iterations required slightly decreases to the range between 30 and 40.

For q-value equal to 0, optimal number of hidden neurons are relatively high (10–13 neurons for relatively balanced ratio of classes up 13–15 neurons for imbalanced ratio of classes equal to 0.3) comparing to other q-values. Due to the complexity of the problem for highly imbalanced datasets for all q-values, the optimal number of hidden neurons is between 4 and 13. For more balanced ratio of classes the number of required neurons is between 1 and 4.

**Figure 7:**
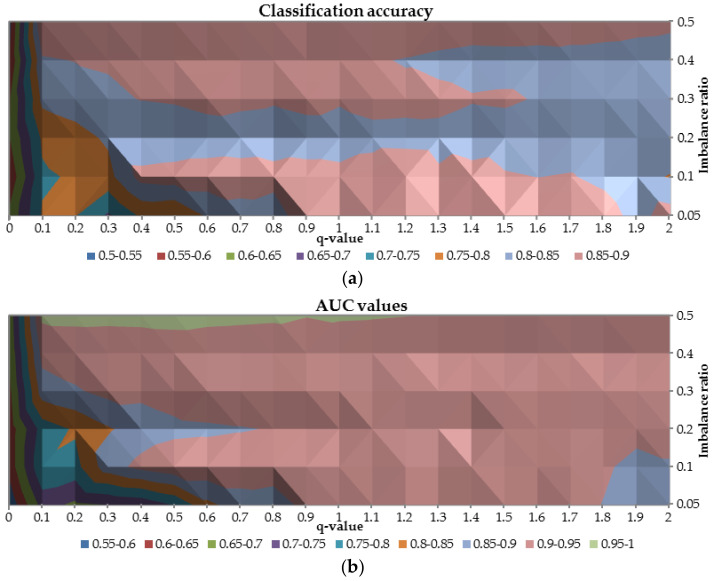
The relation between classification accuracy (**a**) and AUC (**b**) on the test sample in terms of different q-values and class imbalance ratio.

To analyze the overall performance of the ANN trained with q-entropy the AUC and classification accuracy were plotted in Figure 7. Combination of these two metrics provides a general diagnosis of the neural networks’ performance. Once again, the worst results are obtained for q-value equal to 0 for both considered measures (both measures are not higher than 0.65). Between 0.1 and 0.3 for q-value and for class imbalance ratio greater than 0.2, both AUC and classification accuracy are in the range 0.7–0.85, resulted in not acceptable predictive power. It is observed that the highest AUC (>0.90) and the highest classification accuracy (>0.8) were reached when the q-parameter was greater than 0.6.

#### 6.3.2. Results for Different Values of w Parameter

This kind of analysis examines different characteristics of neural networks plotting q-parameter against w coefficient. The class imbalance ratio was set at 0.05 (it is valid for all cases in this subsection) since such ratio is commonly observed in real life applications and it is the most complex in terms of different features [33,34]. The part (**a**) of the Figure 7 presents the relation between q-parameter and neural network capacity to reach convergence in terms of the training function maximization (19) for different values of the w parameter.

Less than 10 iterations are required to train the network when the q-parameter is smaller than 0.3 for all values of the w parameter and for all q-values while the w coefficient reach the highest possible value equal to 1. When the q is greater than 0.6 and w is less than 0.7 the number of required iterations is mostly in the range 30–50. In those cases the network is more focused on the good performance on the validation sample rather than good stability in terms of difference between AUC values on train and validation dataset.

**Figure 8:**
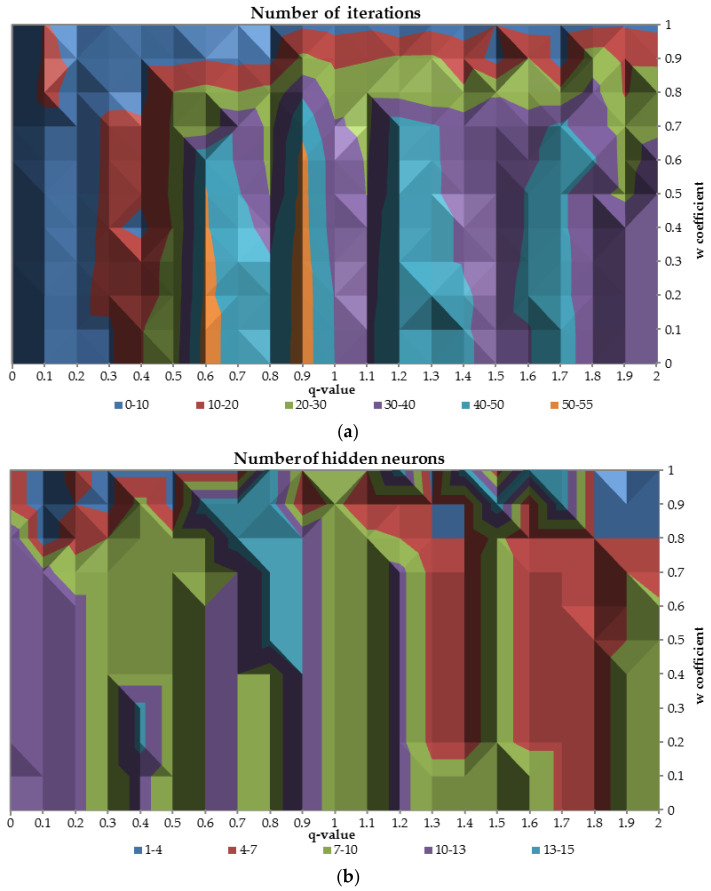
The relation between the number of iterations (**a**) and the number of hidden neurons (**b**) in terms of different values of the q-parameter and the w coefficient.

The part (**b**) of the Figure 8 presents the optimal number of the hidden neurons for the relation between q-parameter and w parameter. On the part (**a**) of the figure (for w greater than 0.8) there is an area where the number of the hidden neurons divers drastically for different values of w coefficient. For the q-values between 0 and 1.3 optimal number of the hidden neurons are in the range 7–13 and for greater values of the q-parameter number of the hidden neurons slightly decrease to the range 4–10, while w parameter is less than 0.8.

**Figure 9:**
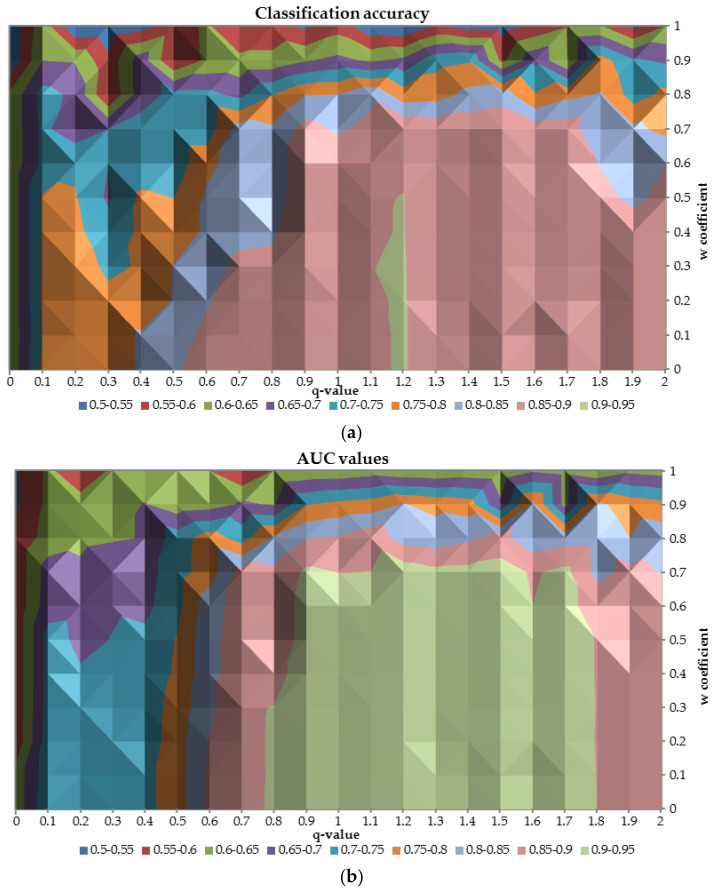
The relation between classification accuracy (**a**) and AUC (**b**) on the test sample in terms of different values of the q-parameter and the w coefficient.

In order to analyze the overall performance of neural networks built for different values of the q-parameter, Figure 9 with AUC and classification accuracy was prepared. The relation between classification accuracy and AUC on the test sample in terms of different values of the q-parameter and the w coefficient is very similar. As shown in the figure the relation is diagonal from the left upper corner to the right lower corner. The best performance neural network achieves when q is greater than 0.8 and w is less than 0.7. When q-parameter decreases and w coefficient increases the overall performance deteriorates up to 0.5 for both measures. Additionally, it was observed that with *q* = 1 the performance of the network was satisfactory, however, there were some areas of its enhanced performance due to application of q measure. For instance, when the q was equal to 1.2 and the w was lower than 0.5 then the classification accuracy was reaching 0.95.

## 7. Conclusions

The article reports the application of the artificial neural network with new entropy cost function to explore its properties on the simulated dataset under different data conditions. The results are considered significant due to following reasons. Firstly, the contribution of this study provides the proof that q-entropy can substitute other standard entropic error functions like Shannon’s one. Secondly, the results of this study indicate that q-entropy error function can be successfully applied in neural networks yielding satisfactory results in terms of both, the number of hidden neurons and number of iterations required to properly train the network, not mentioning the overall performance accuracy. Thirdly, the analysis on simulated dataset comprehensively confirmed that the choice of the error function is indeed an important factor to be examined with great care when designing a neural network for a specific classification problem. In general, the best results were obtained for broad range of q-values, depending on dataset structure. With q-entropy a great flexibility is achieved what enables to derive a final network that exhibits robust performance.

Possible future research on this topic may pertain to the following streams. Firstly, a comparative study to explore the impact of various error functions, including mean square error and the mean absolute error, can be considered. Secondly, the effect of the proposed error functions on other types of network architectures, including application on a variety of real datasets, can be proposed.

## Figures and Tables

**Table 1:** Confusion matrix for binary classification.

		Predicted Value	
		Positive (P)	Negative (N)
Real value	Positive (P)	True positive (TP)	False negative (FN)
	Negative (N)	False positive (FP)	True negative (TN)
